# The Effect of Celecoxib, a Cyclooxygenase-2 Inhibitor on Noise- Induced Hearing Loss

**Published:** 2013-05

**Authors:** Akram Pourbakht

**Affiliations:** Department of Audiology, Faculty of Rehabilitation, Rehabilitation Research Center, Iran University of Medical Sciences, Tehran, Iran

**Keywords:** Auditory brainstem responses, Celecoxib, Guinea pigs, Hair cell, Threshold shift, Noise-induced hearing loss

## Abstract

***Objective(s): ***Noise-induced hearing loss (NIHL) is the major cause of acquired hearing loss. Celecoxib, a cyclooxygenase-2 (COX-2) inhibitor, is a non- steroidal anti- inflammatory drug (NSAID) with known antioxidant and antineoplastic activity. Therefore, we monitored the extent of temporary noise- induced threshold shifts (TTS) and cochlear damage caused by high level 4- kHz noise exposure to verify the differences with those pretreated with celecoxib.

***Materials and Methods:*** Ten male albino guinea pigs (300-350 g in weight) were randomly allocated into two groups: the primal group was exposed to 4- kHz octave band noise at 102 dB SPL for 3 hrs (group 1, n=5); the latter pretreated with 50 mg/ kg celecoxib for 3 days, then exposed to noise (group 2, n=5). Before exposure and one hr after noise exposure, threshold shifts were evaluated with auditory brainstem responses (ABR) and finally the animals were euthanized for histological evaluation.

***Results: ***Comparing the threshold shifts before/after noise exposure with those pretreated, we found out that TTS caused by noise exposure did not show significant mitigation by celecoxib. By observing the organ of Corti at lower middle turn of cochlea in celecoxib pretreated group, considerable hair cell loss was discovered.

***Conclusion:*** The current study clearly confirmed that celecoxib had no attenuation against temporary noise-induced hearing loss.

## Introduction

Noise-induced hearing loss (NIHL) is the most widespread vocational insufficiency in the world. It is stated that about 10 million people are suffering from NIHL in their workplaces, however owing to vicinity to loud music, hunting or traffic noise there are more people at risk. Exposure to chemicals is another factor that exacerbates hearing loss at workplaces. Therefore researchers’ interest for finding a solution to protect cochlea against NIHL is swelling. To prevent hair cell loss against noise, the fundamental biochemical mechanisms that underlie such injury should be comprehended.

Reactive oxygen species (ROS) and reactive nitrogen species (RNS) are known to play an important role in NIHL (1-3). Superoxide anion (O_2_^–•^), hydroxyl radical (^•^OH), hydrogen peroxide (H_2_O_2_) are examples of highly reactive oxygen species that constitute a mechanism for cochlear damage due to high noise level. The nitric oxide (NO) generated after acoustic trauma reacts with superoxide anion (O_2_^•^) forming peroxynitrite (ONOO^-^)

, which is more potent and long-living oxidant (4). In fact, ROS and RNS production exceeding intrinsic antioxidant defense systems leads to apoptosis through participation in the arachidonic acid cascade. Arachidonic acid is produced from membrane phospholipid precursors via phospholipase A2. It generates prostaglandins (PGs) and leukotriens (LTs) via two main pathways: cyclooxygenase (COX) pathways which produce PGs and tromboxane (TXs) and lipooxygenase (LOX) pathway for which LTs are main product. It has been stated that NO induces apoptosis through upregulation of COX-2 expression (5). 

Celecoxib (4-[5-(4-methylphenyl)-3-(trifluoromethyl) pyrazol-1-yl] benzene sulphonimide) is a non-steroidal anti-inflammatory (NSAID) drug which selectively inhibits cyclo-oxygenase (COX-2). COXs are classified into two main isoenzymes: COX-1 and COX-2 (6). COX-1 is a constitutively expressed enzyme found in most tissues, but COX-2 is an enzyme predominantly induced by cytokines, bacterial lipopolysacharides and growth factors (7).

 A homogenous distribution of COX-1 is abundant within nearly all cell types of the organ of Corti, but no COX-1 expression in the cuticular plates of pillar cells has been already revealed. COX-2 has expressed in all cell types, while Hensen cells, neighboring Deiters cells and cuticular plates of outer hair cells showed much stronger expression. Both COX-1 and COX-2 immunoreactions has also been found in spiral ganglion (8). 

Previous studies indicated that NSAIDs could be used as a potential and new treatment strategy for NIHL (9). Similar to other NSAIDs, antioxidant activity for celecoxib has been reported. It is implemented by inhibiting nitric oxide production in osteoarthritis in rat (10) and human (11). Considering such mechanism, it has been approved by US Food and Drug Administration on account for treatment of osteoarthritis and rheumatoid arthritis (12- 13). Besides its antioxidant effect, preclinical studies demonstrated promising antineoplastic activities for celecoxib (14). Importantly, among the medicines of the coxib drug family, the potent pro- apoptotic activity seems to be confined into celecoxib (15). Thus, it has been approved for oral use in prevention of colon cancer development of patients with familiary adenomatous polyposis (FAP) and its clinical therapeutic anti- tumor activity for induction of apoptosis that is widely utilized for treatment of pancreatic, breast, ovarian, non small cell lung cancer (16). Since its clinical use is increasing and considering its bifunctional action (antioxidant or cytotoxic agent), the effect of celecoxib on NIHL must be clarified as well. Therefore, the present study was designed to evaluate the effect of celecoxib, on hearing sufficiency by means of electrophysiological and histopathological study.

## Materials and Methods

***Animals***

Ten healthy, otomicroscopically normal, male albino guinea pigs (Pasteur Institute, Iran) with normal Preyer’s reflex weighing 250- 300 g were selected as experimental subjects for investigation. Animals were randomly distributed into two groups and kept in different cages. Five animals from each group were selected. Animals in group 1 were exposed to 4 kHz octave band noise at 102 dB SPL for 3 hrs without pretreatment (Control). In group 2, subjects pretreated with celecoxib and were later exposed to noise. Celecoxib were given 50 mg/kg (17) dissolved in normal saline and were orally fed by gavage. Animals in group 1 were given saline solution orally by gavage. Celecoxib and saline were administered on a daily basis for 3 successive days. Guinea pigs in groups 1 and 2 were exposed to noise one hr after the final celecoxib or normal saline dose. An hr after exposure, ABR thresholds were measured and compared with baseline thresholds to obtain threshold shift. Then, the animals were euthanized under deep anesthesia and their temporal bones were prepared for histological analysis of organ of Corti damage.

Animals properly anesthetized during auditory brainstem measures. Veterinary care provided by the Unit for Laboratory Animal Medicine of the Faculty of Rehabilitation who are in charge of supervision and provision of animal care and housing.

***Noise exposure***

All exposures were carried out in an illuminated, ventilated and sound-proof chamber, installed with two wire-mesh cages located in corners. One animal per cage was left in exposure and the guinea pigs were allowed to move freely with access to water and food during exposure. The sound chamber was fitted with speakers centrally hanged from the roof and driven by a noise generator and power amplifier. A 0.5 inch Bruel and Kjaer condenser microphone and Fast Fourier Transform analyzer were used to calibrate and measure sound levels at corners within the sound chamber to ensure stimulus uniformity of ± 1 dB within the exposure area.

***Auditory brainstem measurement***

The hearing sensitivity of the animals was evaluated with ABR measurement technique using click stimulus (Eclips EP 25, Denmark). Before measurement, animals were anesthetized with an intramuscular injection of xylazine (4 mg/kg) and ketamine (40 mg/kg). The animals were placed in a sound-proof, electrically isolated room. An inverting needle electrode was placed subcutaneously below the test ear. A needle was placed at the vertex and used as a non- inverting electrode. A ground electrode was also positioned on its back. Wire from each electrode was relayed to an amplifier and then to a custom-designed amplifier. The sound intensity was initially varied in 10-20 dB steps from 80 dB SPL and in 5 dB steps near the threshold. Hearing threshold was visually interpolated between the last repeatable appearance of a wave 3- 4 and 5 dB less where no peak is evident in the ABR. 

***Histological examination***

The guinea pigs were sacrificed after the last ABR measurement performed by decapitation under deep anesthesia by a mixture of xylazine and ketamine. Temporal bones were immediately removed, the round and oval windows were exposed and the perilymphatic spaces perfused with 2% paraformaldehyde in 0.1 M phosphate buffer at pH 7.4 for 1 hr, then washed in 0.1 M phosphate buffer. The bony shell of the cochlea were removed, and the specimens were soaked in 0.3% Triton X-100 for 5 min to make the hair cell membranes permeable and then whole mounts of the organ of Corti were stained for F-actin for 60 min with rhodamine phalloidin. After staining and under a dissecting microscope, tectorial membrane were removed and the osseous spiral lamina partially dissected away, and lower turns of the organ of Corti mounted on glass slides. Slides were observed under a fluorescence microscope at a magnification of 40*×*. 

***Statistics***

Sigma Stat statistical software was used. The Kolmogorov- Smirnov test (K- S test) was used to assess the data normality in each group. Threshold shifts at each group were compared by t- test. *P*< 0.05 were considered statistically significant.

## Results

The hearing thresholds before noise exposure were equivalently dispatched for all ears (mean 13.3±2.8 dB SPL). There were no significant differences among groups. The results of ABR thresholds before and an hr after noise exposure in each group is displayed in figure 1.

In control group, the average of auditory threshold in one hr post- noise exposure was 17.75 ± 4.78 dB SPL. It was significantly higher when was compared to pre- noise exposure (*P* < 0.05). This result showed that there was considerable threshold shift after noise exposure. In the group receiving celecoxib before noise exposure, the average of auditory threshold in one hr post- noise exposure was 22.5±2.8 dB SPL. It was significantly higher when compared to pre- noise exposure (*P* < 0.05). This result showed that there was considerable noise- induced threshold shift after celecoxib pretreatment. After comparing the ABR threshold shifts between groups, it proved that celecoxib pretreated group had higher threshold shift than control. This difference was not statistically significant. The current study's results indicated that celecoxib could not attenuate temporary noise- induced threshold shift.

Rhodamine Phalloidin staining was used to study cochlear damage after noise exposure. Inner hair cells (IHCs) and supporting cells after noise exposure seemed to remain normal. However, outer hair cells (OHCs) were fairly injured. The stereocillia in remaining outer hair cells formed a W-shape arrangement, while stereocillia on inner hair cells (IHC) were sorted in U- shape (Figure 2). Figure 3 shows the condition of hair cell in the lower middle turn of the organ of Corti after noise exposure pretreated with celecoxib. Some damage on outer hair cells observed, while inner hair cells and supporting cells were not injured. In both groups, cells with an identifiable cell body and cuticular plate were found salubrious. The streocilia in remaining cells also appeared to be normal. We observed distinctive scar formation in OHCs produced by convergence of adjacent phalangeal processes of supporting cells. Supporting cell adjacent to degenerating OHCs expand and form a permanent epithelial scar which maintains the integrity of the reticular lamina. This histological view is regarded to as a sign of hair cell loss. These results concluded that 50 mg/ kg celecoxib has no significant hair cell protection when using 4 kHz noise.

## Discussion

In current study, we evaluated whether 50 mg/ kg celecoxib, could reduce noise- induced temporary threshold shift and cochlear damage by means of ABR measurement and histological observation. Our electrophysiological results showed that pretreatment with celecoxib did not significantly reduce ABR threshold shift using click stimulus an hr after 4 kHz octave band noise at 102 dB SPL for 3 hrs. The light microscopic observation also showed damage to the organ of Corti at the lower middle turn of the cochlea in pretreated group. The results of the current study concluded that celecoxib could not attenuate noise- induced TTS and cochlear damage.

It is speculated that 4 kHz octave band noise has a maximum energy around 3-6 kHz, reducing at least 30 dB SPL in upper and lower frequency areas (18). There are two patterns of threshold shift and cochlear damage after noise exposure, which is related to basilar membrane response, acoustic characteristics of noise and temporary effect of it. Threshold shift after noise exposure either recovers to baseline level (temporary threshold shift, TTS) or some permanent shift (permanent threshold shift, PTS) remains. In our study, exposure to 4 kHz octave band of noise in control group caused considerable TTS. We confirmed this threshold shift as a TTS according to our previous lab's study (submitted article). In that study we used the same noise parameters (4 kHz octave band noise at 102 dB SPL for 3 hrs) and measured ABR an hr and one week later. We proved that threshold shift an hr after noise exposure returned to baseline level a week later. Therefore, in current study, we had considerable TTS which can be explained by maximal energy level of noise used in control group.

Excessive production of ROS and/ or RNS appears to injure hair cells following exposure to noise. It has been shown that hair cell survival increases after ROS and/ or RNS scavenging or by enhancing intrinsic antioxidants such as glutathione (19). Administration of extrinsic antioxidants including N- acetyl- cysteine (L-NAC), D-methionine and ebselen reduces NIHL (20- 21). L-NAC and D- methionine are precursors of glutathione that interact with hydroxyl groups and peroxides (H2O2). Ebselen is a glutathione peroxidase mimic that scavenges peroxynitrate. Ajith *et al* (2005) showed an antioxidant activity for celecoxib (22). It has been speculated that celecoxib inhibits nitric oxide production in osteoarthritis in rat (10) and in human (11). Hence, in current study, animals were given celecoxib before noise exposure to evaluate their prophylactic effect against NIHL. However, considerable noise- induced threshold shift remained after celecoxib pretreatment. This result demonstrated that there was no TTS attenuation after celecoxib administration.

Each of cochlear cellular systems such as hair cells, nervous system, supporting cells and even circulation system are vulnerable to damage after noise exposure. In the beginning outer hair cells injures, and if the intensity or duration of exposure increases, damage spreads to inner and supporting cells. The cochlear blood flow is sensitive to blood level of carbon dioxide, nitric oxide, prostaglandins and tropomyosin. The role of prostaglandins as a product of COX enzyme in cochlea is known for years. PGE2 and PGI2 increase inner ear blood flow (23). Vascular constriction results in decreased cochlear blood flow and has been reported after noise trauma via PGF2α (24). Previous studies showed there is COX-2 enzyme in the organ of Corti and cochlear spiral ganglion (8), but still its role in inner ear physiology is not well known. In present research, we studied the effect of COX-2 on hearing by means of its selective inhibitor, celecoxib. We observed an approximate area around lower middle turn, which is compatible with most damaged area caused by 4 kHz octave band of noise, under fluorescent microscope. We observed that outer hair cells were more vulnerable than inner hair cells and OHC damage caused by noise could not be inhibited by celecoxib. This result showed that celecoxib has no protective effect on the organ of Corti at the area affected by noise trauma.

Analgesics such as NSAIDs are clinically at practice widely. There are few reported studies on the protective effects of diclofenac (25) and salicylate (20) on NIHL. There is however one study on the literature showed that COX-2 inhibitors, meloxicam, SC58125, and CAY 10404 had no significant effect on ABR threshold shift (9). They administered COX-2 inhibitors post- noise exposure and evaluated their effects on PTS and finally, concluded that NSAIDs could be used as a potential and new treatment strategy for cochlear injury. But among NSAIDs and specifically Coxib drug family, it demonstrated that celecoxib has a bifunctional effect. It induces apoptosis related to its antineoplastic properties (14). Celecoxib targets several proteins distinct from COX- 2 that are involved in control of cell survival and cell death. In COX-2 expressing cells, inhibition of COX-2 may contribute to cytotoxic effects. The possible mechanisms of cochlear protection by NSAIDs are their anti-inflammatory and antioxidant actions. A study (2001) using celecoxib (10, 50, 100, and 200 mg/kg) in rat showed that anti-inflammatory efficacy of celecoxib (statistically significant at 50 mg/ kg) is completely lost at doses of 100- 200 mg/ kg (17). Thus, we found dose of 50 mg/kg compatible with our study objectives. In current study's electrophysiological data, celecoxib had a higher TTS than control group. Of course such difference was not statistically significant; however considering OHC damage observed, an ototoxic effect should be taken into account. It may be possible to obtain an ototoxic effect using celecoxib at higher doses or longer duration.

In this study, we used click stimulus which covers part of cochlea compatible with most damaged area by 4 kHz noise exposure. However, ototoxic damage to cochlea begins in the base and spreads to the apex. The base to apex damage gradients is caused because of intrinsic antioxidant level differences (26) and the uptake of ototoxic medicine (27). There might be some early damage at the base of cochlea which could not be detected by the stimulus used in this study. The technical limitation of current study in using click stimulus in ABR measurement and single dose of celecoxib should be considered and clarifies the need for further study by high frequency stimulus and different doses of medicine.

## Conclusion

In current study, celecoxib induced moderate temporary threshold shifts and showed some hair cell loss compared to control group. Therefore, we concluded that celecoxib, had no significant attenuation against temporary noise- induced threshold shift and hair cell loss. 

## References

[1] Yamane H, Nakai Y, Takayama M, Iguchi H, Nakagawa T, Kojima A (1995). Appearance of free radicals in the guinea pig inner ear after noise-induced acoustic trauma. Eur Arch Otorhinolaryngol.

[2] Ohlemiller KK, Wright JS, Dugan LL (1999). Early elevation of cochlear reactive oxygen species following noise exposure. Audiol Neurootol.

[3] Shi X, Ren T, Nuttall AL (2002). The electrochemical and fluorescence detection of nitric oxide in the cochlea and its increase following loud sound. Hear Res.

[4] Arteel GE, Briviba K, Sies H (1999). Protection against peroxynitrite. FEBS Lett.

[5] Li MH, Jang JH, Surh YJ (2005). Nitric oxide induces apoptosis via AP-1-driven upregulation of COX-2 in rat pheochromocytoma cells. Free Radic Biol Med.

[6] Gierse JK, McDonald JJ, Hauser SD, Rangwala SH, Koboldt CM, Seibert K (1996). A single amino acid differences between cyclooxygenase-1 (COX-1) and -2 (COX-2) reverses the selectivirty of COX-2 specific inhibitors. J Biol Chem.

[7] Barnett J, Chow J, Ives D, Chiou M, Mackenzie R, Osen E (1994). Purification, characterization and selective inhibition of human prostaglandin G/H synthase 1 and 2 expressed in the baculivirus system. Biochem Biophys Acta.

[8] Ziegler EA, Brieger J, Heinrich UR, Mann WJ, Chambers HF, Sande MA, Hardman JG, Limbird LE, Molinoff PB, Ruddon RW, Gilman AG (1996). Goodman and Gilman’s, The Pharmacological Basis of Therapeutics.

[9] Hoshino T, Tabuchi K, Hirose Y, Uemaetomari I, Murashita H, Tobita T (2008). The non- steroidal anti- inflammatpry drugs protects mouse cochlea against acoustic injury. Tohoku J Exp Med.

[10] Matsuda K, Nakamura S, Matsushita T (2006). Celecoxib inhibits nitric oxide production in chondrocytes of ligament-damaged osteoarthritic rat joints. Rheumatol Int.

[11] Ozgocmen S, Ardicoglu O, Erdogan H, Fadillioglu E, Gudul H (2005). In vivo effect of celecoxib and tenoxicam on oxidant/ anti-oxidant status of patients with knee osteoarthritis. Ann Clin Lab Sci.

[12] Penning TD, Talky JJ, Bertenshaw JR (1997). Synthesis and biological evaluation of the 1,5-dioxy pyrazol class of cyclooxygenase inhibitors: Identification of 4-[5-1,4-methyl phenyl 3-trifluromethyl-III-pyrazole-1-yl] benzene sulfonimide (SC-56635 celecoxib). J Med Chem.

[13] Simon LS, Laza FL, Lipsky PE (1998). Preliminary study of the safety and efficacy of sc 56635, a novel cyclooxygenase 2 inhibitor. Efficacy and safety in two placebo-controlled trials in osteoarthritis and rheumatoid arthritis, and studies of gastrointestinal and platelet effects. Arthritis Rheum.

[14] Jendrossek V (2011). Targeting apoptosis pathways by celecoxib in cancer. Cancer Lett.

[15] Schiffmann S, Maier TJ, Wobst I, Janssen A, Corban- Wilhelm H, Angioni C (2008). The anti- proliferative potency of celecoxib is not a class effect of coxibs. Biochem Pharmacol.

[16] Arber N, Eagle CJ, Spicak J, Racz I, Dite P, Hajer J, et- al (2006). Celecoxib for the prevention of colorectal adenomatous polyps. N Engl J Med.

[17] Niederberger E, Tegeder I, Vetter G, Schmidtko A, Schmidt H, Euchenhoffer C (2001). Celecoxib loses its anti- inflammatory efficacy at high doses through activation of NF- kB. FASEB J.

[18] Pourbakht A, Yamasoba T (2003). Cochlear damage caused by continuos and intermittent noise exposure. Hear Res.

[19] Raphael Y (1991). Altschuler RA. Scar formation after drug-induced cochlear insult. Hear Res.

[20] Harding G W, Bohne BA (2009). Relation of focal hair-cell lesions to noise-exposure parameters from a 4- or a 0. 5-kHz octave band of noise. Hear Res.

[21] Yamasoba T, Nuttall AL, Harris C, Raphael Y, Miller JM (2008). Role of glutathione in protection against noise-induced hearing loss. Brain Res.

[22] Kopke RD, Weisskopf PA, Boone JL, Jackson RL, Wester DL, Hoffer ME (2000). Reduction of noise- induced hearing loss using L- Nac and salicylates in the chinchilla. Hear Res.

[23] Pourbakht A, Yamasoba T (2003). Ebselen attenuates cochlear damage caused by acoustic trauma. Hear Res.

[24] Ajith TA, Sanjay PS, Babitha NV (2000). Antimutagenic and anti-oxidant activities of the non-steroidal anti-inflammatory drug celecoxib. Clin Exp Pharmacol Physiol.

[25] Rhee CK, Park YS, Jung TTK, Park CI (1999). Effects of leukotriens and prostaglandins on cochlear blood flow in the chinchilla. Eur Arch Otorhinolaryngol.

[26] Miller JM, Brown JN, Schacht J (2003). 8- Iso- prostaglandin F2 alfa, a product of noise exposure, reduces inner ear blood flow. Audiol Neurootol.

[27] Lamm K, Arnold W (1998). The effect of prednisolone and non- steroidal anti- inflammatory agents on the normal and noise- damaged guinea pig inner ear. Hear Res.

[28] Sha SH, Taylor R, Forge A, Schacht J (1999). Differential vulnerability of basal and apical hair cells is based on intrinsic susceptibility to free radicals. Hear Res.

[29] Dai CF, Steygerm PS (2008). A systemic gentamycin pathway across the stria vascularis. Hear Res.

